# Highly sensitive ammonia sensor for diagnostic purpose using reduced graphene oxide and conductive polymer

**DOI:** 10.1038/s41598-018-36468-z

**Published:** 2018-12-21

**Authors:** Tan Nhiem Ly, Sangkwon Park

**Affiliations:** Department of Chemical and Biochemical Engineering, Dongguk University, Pildong-ro 1-gil 30, Jung-gu, Seoul, 04620 South Korea

## Abstract

In this study, we fabricate ammonia sensors based on hybrid thin films of reduced graphene oxide (RGO) and conducting polymers using the Langmuir-Schaefer (LS) technique. The RGO is first prepared using hydrazine (Hy) and/or pyrrole (Py) as the reducing agents, and the resulting pyrrole-reduced RGO (Py-RGO) is then hybridized with polyaniline (PANI) and/or polypyrrole (PPy) by *in-situ* polymerization. The four different thin films of Hy-RGO, Py-RGO, Py-RGO/PANI, and Py-RGO/PPy are deposited on interdigitated microelectrodes by the LS techniques, and their structures are characterized by scanning electron microscopy (SEM) and atomic force microscopy (AFM). The results of ammonia sensing experiments indicate that the Py-RGO/PANI film exhibits the highest sensor response of these four films, and that it exhibits high reproducibility, high linearity of concentration dependency, and a very low detection limit (0.2 ppm) both in N_2_ and exhaled air environments. The current gas sensor, therefore, has potential for diagnostic purposes because it has the additional advantages of facile fabrication, ease of use at room temperature, and portability compared to conventional high-sensitivity ammonia sensors.

## Introduction

In the last decade, mobile devices such as personal digital assistants (PDAs), smartphones, and tablet computers have been employed in healthcare systems. This is typically referred to as mobile health (m-health) or ubiquitous health (u-health), and it has led to fundamentally new ways of healthcare management^1–3^. In addition, smart wearable^4,5^, and implantable systems^6,7^, such as sensors, actuators, wireless communication networks, and other devices have recently been developed for m-health and/or u-health applications. These systems are capable of not only monitoring the general health status of patients by providing measurements of their physiological and activity signals such as body temperature, heart rate, blood pressure, and motion activity, but they can also provide readouts for specific pathologies such as cardiovascular diseases, diabetes, respiratory diseases, and renal diseases by measurement of vital parameters of electrocardiograms, blood pressure, blood glucose levels, the respiration rate, and the ammonia concentration in breath^4,5,8^.

Although the advent of m-health (or u-health) has increased the accessibility and the affordability of healthcare, there is still room for development of diagnostic sensors. Low-cost, high-quality, easy-to-use, non-invasive, wearable, and high-throughput sensor technology is a fundamental and critical factor for the implementation of advanced m-health^9^. An example of this is renal disease (also referred to as kidney failure), which is one of the most common health issues in the world. Renal disease patients progressively lose the dialysis function of their kidneys. Due to this dysfunction, urea generated by the urea cycle remains in the blood even after passing through the kidney, and it accumulates in blood as ammonium ions that permeate through the lung membranes by the respiratory process as NH_3_ gas. When renal patients exhale, their breath, therefore, usually contains a level of NH_3_ that ranges from 1.5 ppm to 15 ppm^10,11^. In order to monitor and treat the disease early, several conventional techniques including urine testing, blood testing, and biopsy are used to assess a number of indicators of renal disease such as the amount of blood urea nitrogen and the glomerulus filtration rate^12^. However, these techniques are highly invasive and they require skilled personnel at medical centers. Clearly, it would be highly desirable to have a non-invasive and easy-to-use technique for the diagnosis of renal disease at early stage. Thus, a low cost, portable, and easy-to-use sensor that can detect low levels of NH_3_ gas in the exhaled breath of patients would be a particularly useful tool for diagnosis of the disease in the context of m-health.

The literature to date indicates that NH_3_ sensors have been developed using a range of techniques such as laser-coupled spectroscopy^13^, photoacoustic spectroscopy^14^, metal oxide devices^15^, and quartz crystal microbalances^16^. One of the most commonly used types of commercial ammonia sensor is electrochemical sensor, which is based on a gas diffusion cell connected to an electrolyte solution where ammonia gas is detected by subsequent techniques such as amperometry, potentiometry and colorimetry^17–19^. Although various types of aqueous and nonaqueous solutions including ionic liquid and organic gel have been used as the electrolyte solution, a common drawback for this type of sensor is the problem of electrolyte consumption during electrochemical reaction, which makes the sensor’s lifespan limited.

Although these techniques have allowed the desired level of sensitivity and reproducibility to be achieved for their specific purposes, there is still a clear need for a cost-effective, portable, and on-site measurable sensors for use with m-health. Reduced graphene oxide (RGO) and conductive polymers appear to be promising materials for meeting these requirements. Due to its p-type semiconductor behavior in electron transition, RGO has recently seen extensive use as a sensing material in various types of chemical and biochemical sensors including gas sensor^20–22^. In particular, these characteristics of RGO have been used as resistive sensor materials to detect electron donor gases such as NH_3_^21–24^. However, RGO has not been actively applied to detect trace amounts of NH_3_ down to 1.5 ppm with high sensitivity and selectivity. This is thought to be because it is difficult to control its structure and chemical reactivity in sensing films. Recently, conducting polymers have been extensively applied as active sensing layers for gas detection as result of their high sensitivity, short response times, room temperature operation, easy sensor element processing, and light weight. Polyaniline (PANI) and polypyrrole (PPy) in particular have been used most often due to their high chemical stability, ease of electrochemical and chemical synthesis, simple and reversible doping and de-doping chemistry, and stable electrical conduction mechanism^25,26^. Moreover, both PANI and PPy are known to be highly compatible with RGO^27,28^. Hybrid films of RGO combined with PANI or PPy can, therefore, be expected to exhibit a synergistic effect that results in higher sensitivity.

We here report the fabrication and characterization of hybrid thin films of RGO and PANI and/or PPy with well-controlled thicknesses and structures by the Langmuir-Schaefer (LS) self-assembly technique on a substrate of interdigitated microelectrodes (IDEs). First, graphene oxide (GO) was reduced to RGO by hydrazine and/or pyrrole, and the RGO was incorporated with PANI and/or PPy by *in-situ* polymerization. Four different hybrid thin films of RGO and PANI and/or PPy were deposited on the IDEs surfaces, and their NH_3_ sensing performances were measured as a function of the NH_3_ concentration in a nitrogen and/or a simulated exhaled air environment containing carbon dioxide, water vapor, and other trace gases. The sensitivity results for the various thin films were compared and they are discussed in terms of the thin film structures and the gas-sensing mechanism. The potential of the fabricated sensor as a diagnostic tool for renal disease is also discussed.

## Results and Discussion

**Figure 1:**
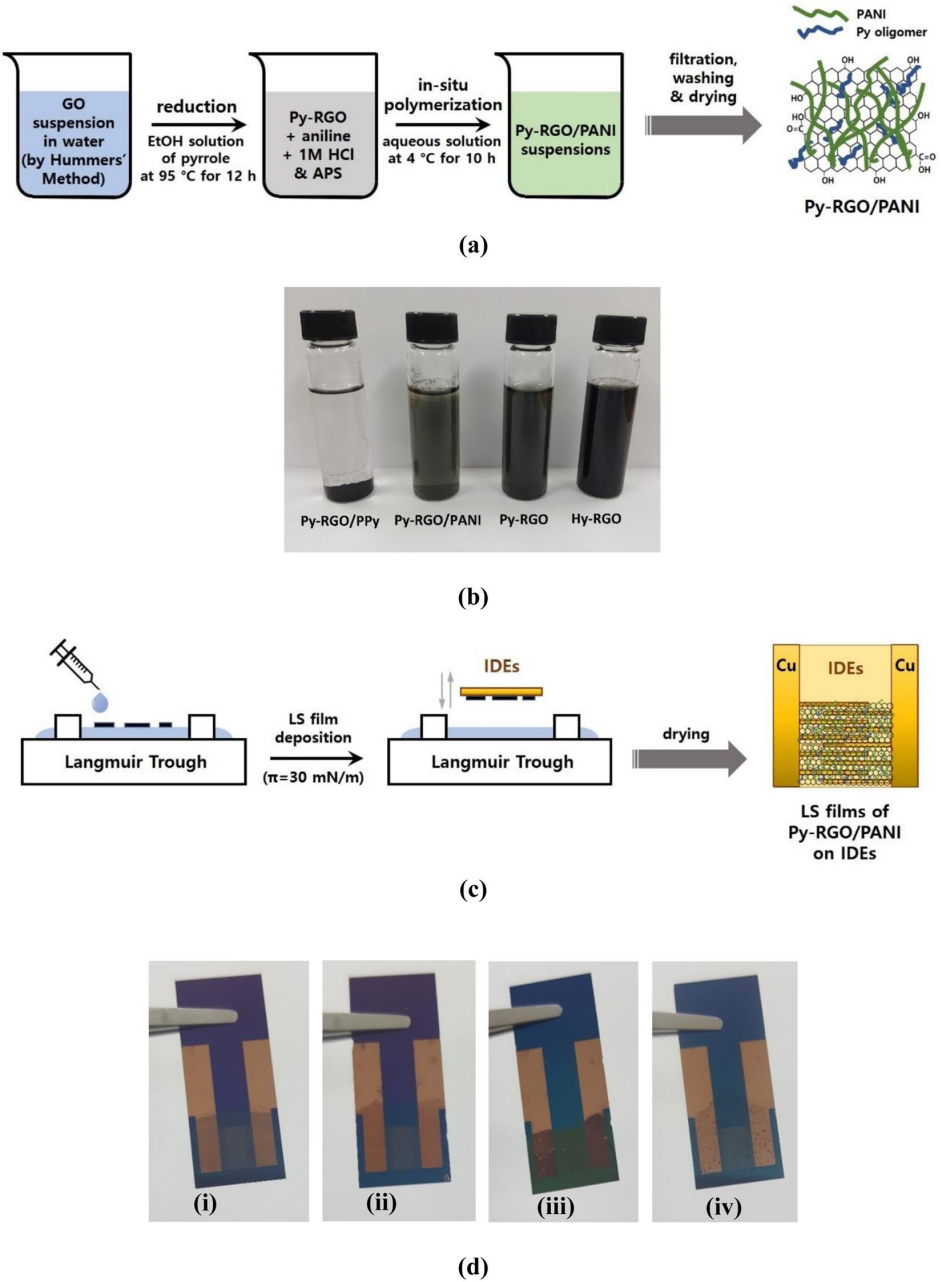
(**a**) Illustration of preparation procedure for Py-RGO/PANI hybrid particles (**b**) suspensions of Hy-RGO (in DMF/DI water), Py-RGO, Py-RGO/PANI, and Py-RGO/PPy (in ethanol) (**c**) illustration of preparation procedure for the LS films of Py-RGO/PANI, and (**d**) actual LS films of Hy-RGO, Py-RGO, Py-RGO/PANI, and Py-RGO/PPy deposited on IDEs.

**Figure 2:**
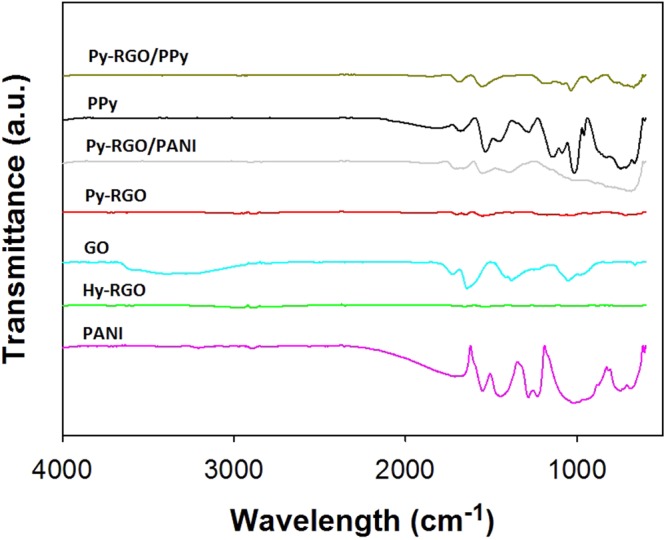
Raman spectra of GO, Hy-RGO, Py-RGO, PANI, Py-RGO/PANI, PPy, and Py-RGO/PPy.

Using the synthetic procedures described in Fig. 1, four different RGO and their hybrid particles with PANI and PPy (i.e., Hy-RGO, Py-RGO, Py-RGO/PANI, and Py-RGO/PPy) were successfully fabricated and characterized using Raman and FT-IR spectroscopy. As shown in Fig. 2, there were no distinct differences between the Raman spectra for GO, Hy-RGO, and Py-RGO, which had two peaks at 1,340 and 1,600 cm^−1^ that correspond to the D and G bands, respectively, that are typical fingerprint bands for RGO^29^. For Py-RGO/PANI, the spectrum had two peaks, at 900 and 1,590 cm^−1^, which are representative peaks for PANI, as well as the peaks at 1,340 and 1,600 cm^−1^ that are representative for Py-RGO^30^. For PPy, there were two peaks at 975 and 1,061 cm^−1^, representing ring deformation and associated dications, respectively^31^. These peaks were also present in the spectrum of Py-RGO/PPy, along with the peaks at 1,340 and 1,600 cm^−1^ for Py-RGO. In addition, XPS and TGA analysis results were given in the Fig. S1 and S2 in the supplementary information. These results indicate that the hybrid particles of Py-RGO/PANI and Py-RGO/PPy were prepared successfully.

**Figure 3:**
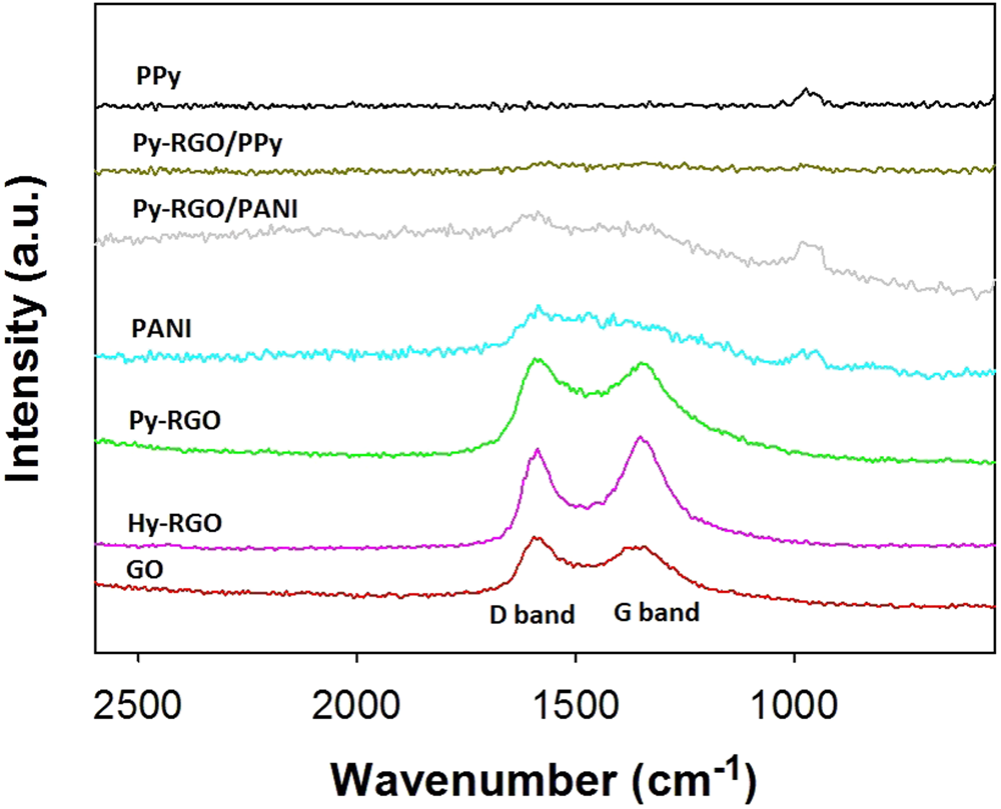
FT-IR spectra of GO, Hy-RGO, Py-RGO, PANI, Py-RGO/PANI, PPy, and Py-RGO/PPy.

The chemical compositions of the fabricated hybrid particles of Py-RGO/PANI and Py-RGO/PPy were also analyzed by FT-IR spectroscopy. As shown in Fig. 3, for GO there were four peaks at 3,399, 1,724.7, 1,641.3 and 1,381.5 cm^−1^, which represent O-H stretching vibrations, C=O stretching vibrations (from carbonyl and carboxylic groups), skeletal vibrations from unoxidized graphitic domains, and C-O stretching vibrations, respectively^32^. The peaks for these functional groups were barely discernible in the spectrum for Hy-RGO, whereas they had reduced amplitude in the spectrum of Py-RGO, thus indicating that the functional groups on the GO surface were almost completely reduced by hydrazine, whereas they were partly reduced by pyrrole. PANI exhibited two peaks at 1,550.9 and 1,444.4 cm^−1^ that correspond to the non-symmetric vibration of C-H bonds, and another two peaks at 1,283.8 and 1,208.4 cm^−1^ that correspond to C-N bond stretching on the quinoid and the benzenoid moiety. It should be noted that the peak at 744.7 cm^−1^ represents a polar structure caused by proton doping^33^. The spectrum of Py-RGO/PANI exhibited peaks for both Py-RGO and PANI at 1,711.3, 1,553.5, 1,390.3 and 690.3 cm^−1^. Similarly, the spectrum for Py-RGO/PPy exhibited the peaks at 1,014, 1,142, 1,554 and 1,679 cm^−1^ found for PPy, of which the peak at 1,554 cm^−1^ is characteristic of C=C stretching and pyrrole ring vibration^31^. These results confirmed the successful fabrication of hybrid particles of Py-RGO/PANI and Py-RGO/PPy.

**Figure 4:**
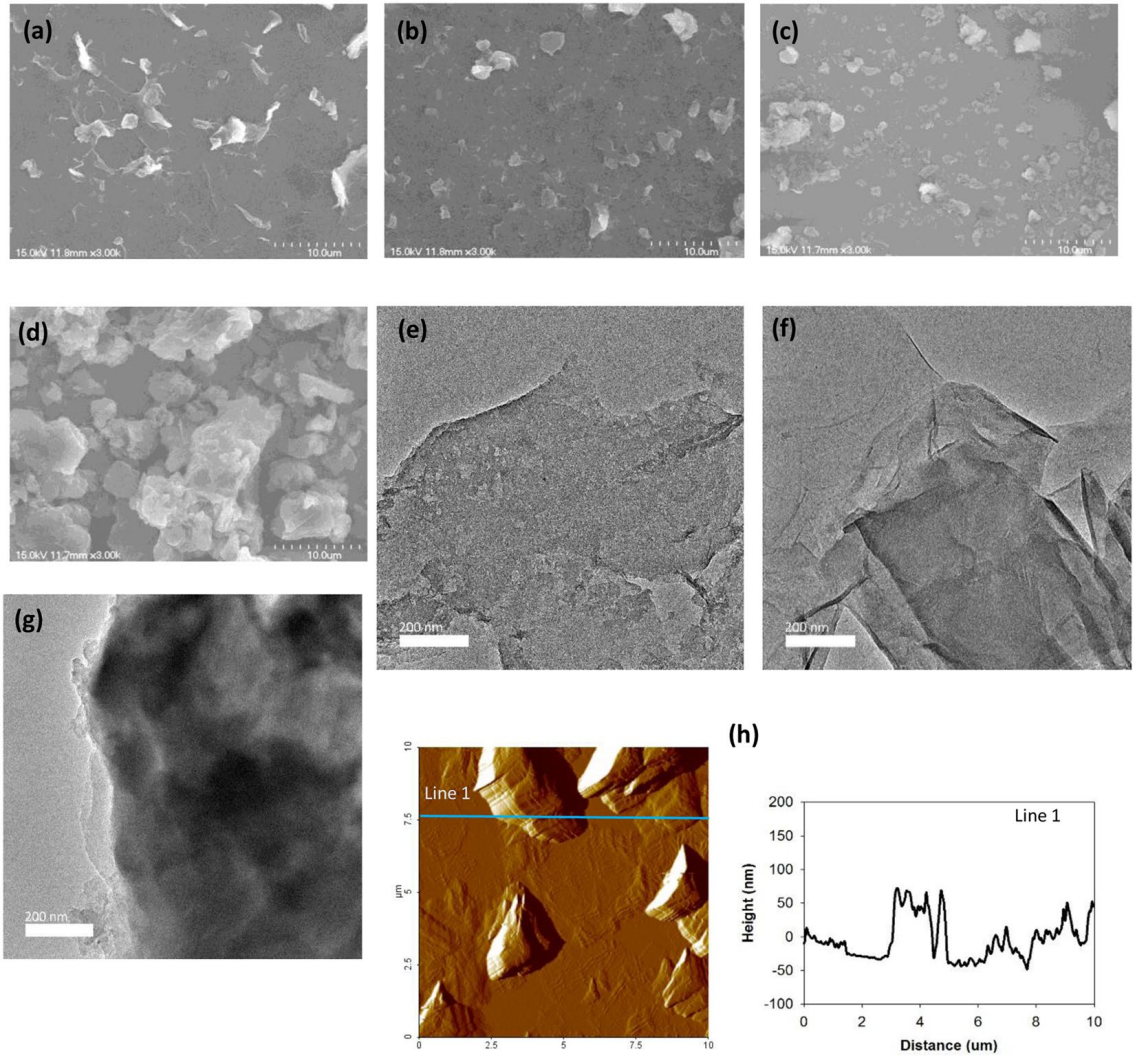
SEM micrographs of the LS films of (**a**) Hy-RGO (**b**) Py-RGO (**c**) Py-RGO/PANI (**d**) Py-RGO/PPy, TEM images of (**e**) Py-RGO (**f**) Py-RGO/PANI (**g**) Py-RGO/PPy and (**h**) AFM topography of Hy-RGO.

For LS film formation, the Py-RGO, Py-RGO/PANI, and Py-RGO/PPy powders were redispersed in ethanol by sonication for 30 min. For LS film formation of Hy-RGO, the as-prepared Hy-RGO suspension in DMF/DI water was used directly because it exhibited long-term stability. As shown in Fig. 1(b), three hours after the sonication for 30 min, the Hy-RGO and the Py-RGO particles maintained highly dispersed states in DMF/DI water and ethanol suspensions, respectively. On the other hand, some of the Py-RGO/PANI particles aggregated, and most of the Py-RGO/PPy particles underwent significant aggregation and precipitated to the bottom of the container, thereby resulting in a transparent ethanol solution. Therefore, the Py-RGO/PANI and the Py-RGO/PPy suspensions were sonicated for an additional 30 min before use. The suspensions of the four different particles were spread at the air/water interface to deposit corresponding LS films on the IDEs. The resulting LS films are shown in Fig. 1(d) and their morphologies are shown in Fig. 4. The Hy-RGO film surface had RGO sheets of a few microns in size that had undergone a considerable degree of folding and aggregation, presumably because the GO sheets were significantly reduced by hydrazine (Hy). On the other hand, the Py-RGO film surface had fewer aggregated and folded RGO sheets, presumably due to a lesser degree of reduction by pyrrole (Py) than by Hy. When the GO sheets were reduced by Py, the Py molecules were oxidized and formed oligomers that were adsorbed by the RGO surface. As the Py oligomers contain a high number of sp^2^ carbon atoms they readily adsorbed to the surface of the Py-RGO by π-π interactions^31^. As shown in TEM images in Fig. 4, the Py-RGO/PANI film surface is apparently similar to that of Py-RGO, but it has dark regions which is presumably due to adsorbed layers of PANI molecules. On the other hand, the Py-RGO/PPy film shows a blurry and darker surface which is presumably due to thick aggregates of PPy. According to a report in the literature, the Py-RGO sheets were adsorbed with homogeneously distributed PANI molecules having fibrous structures of a few hundred nm in length and about 50 nm in width^28^. On the other hand, the Py-RGO/PPy film surface was dominated with large aggregates of a few hundred nm size, which were presumably PPy particles. The same *in-situ* polymerization procedure as in the current study usually produces PPy aggregates with a granular shape and diameters of 200–300 nm and a high degree of porosity^29^. In addition, as shown in Fig. 4(h) the surface roughness of the Hy-RGO LS films was estimated to be about 30.5 nm from the AFM topography, which is much less than the value reported in the literature (about 84.7 nm) for an LS film of RGO prepared from a methanol suspension^34^. This reduced surface roughness can be attributed to the stabilization of RGO sheets by the DMF that was used as the solvent in the current study. DMF engages in polar interaction and hydrogen bonding with RGO that keep the RGO sheets from undergoing severe aggregation in the dispersion.

**Figure 5:**
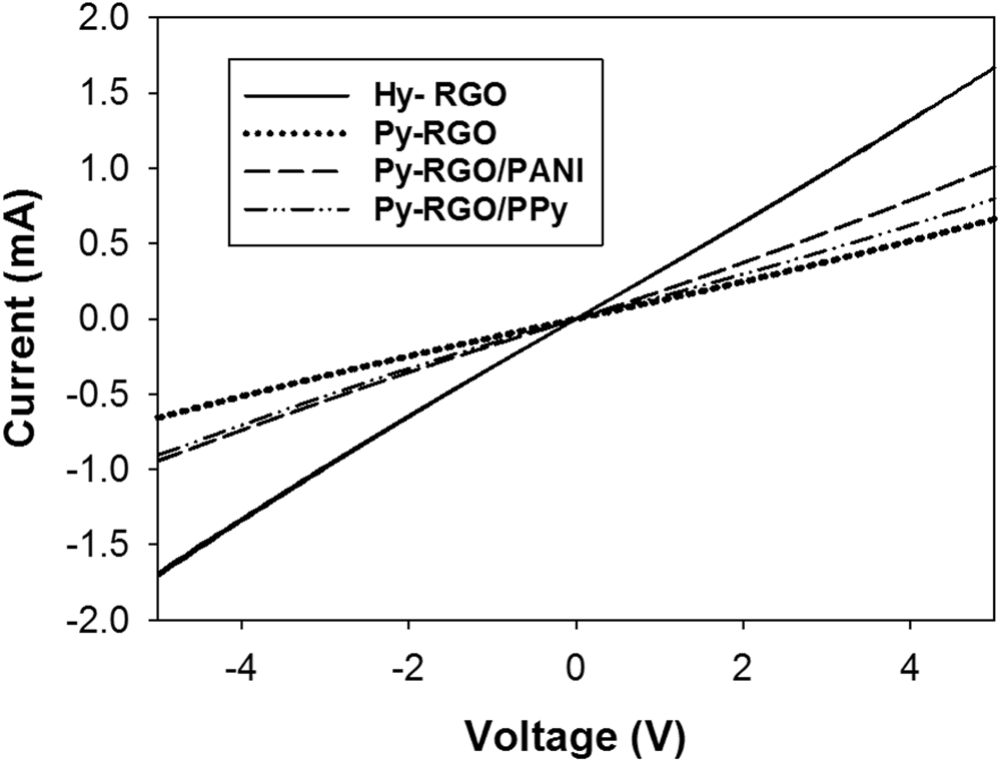
I–V characteristics of the LS films of Hy-RGO, Py-RGO, Py-RGO/PANI, and Py-RGO/PPy deposited on the IDEs.

**Figure 6:**
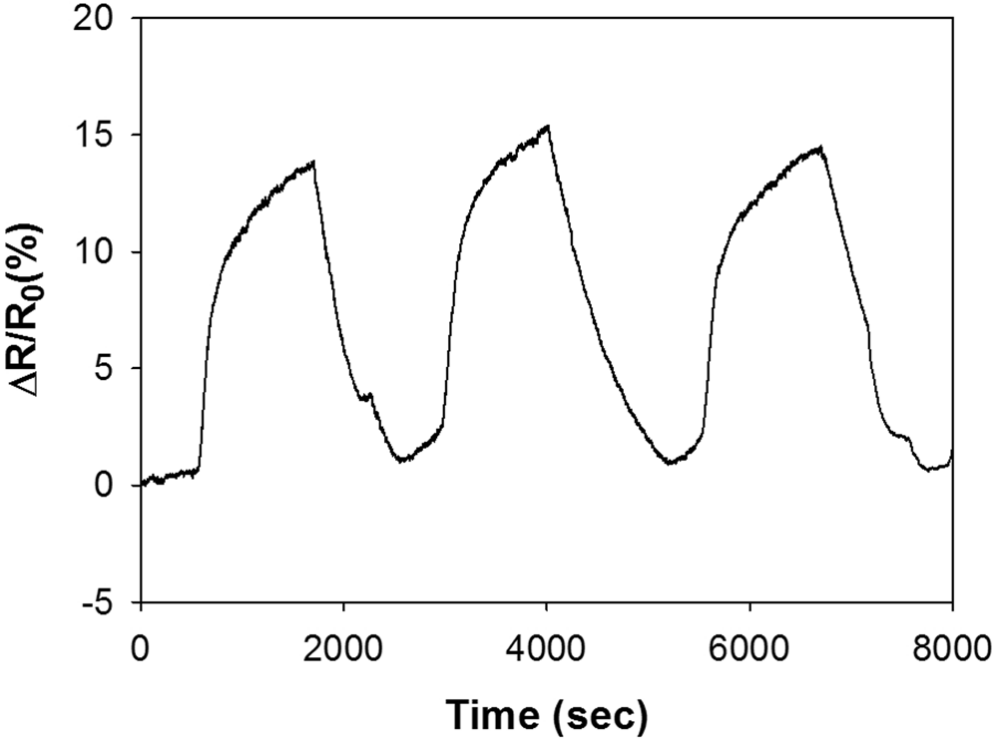
Reproducibility of the sensor response of the Hy-RGO LS films upon exposure to NH_3_ at 50 ppm in a nitrogen environment.

**Figure 7:**
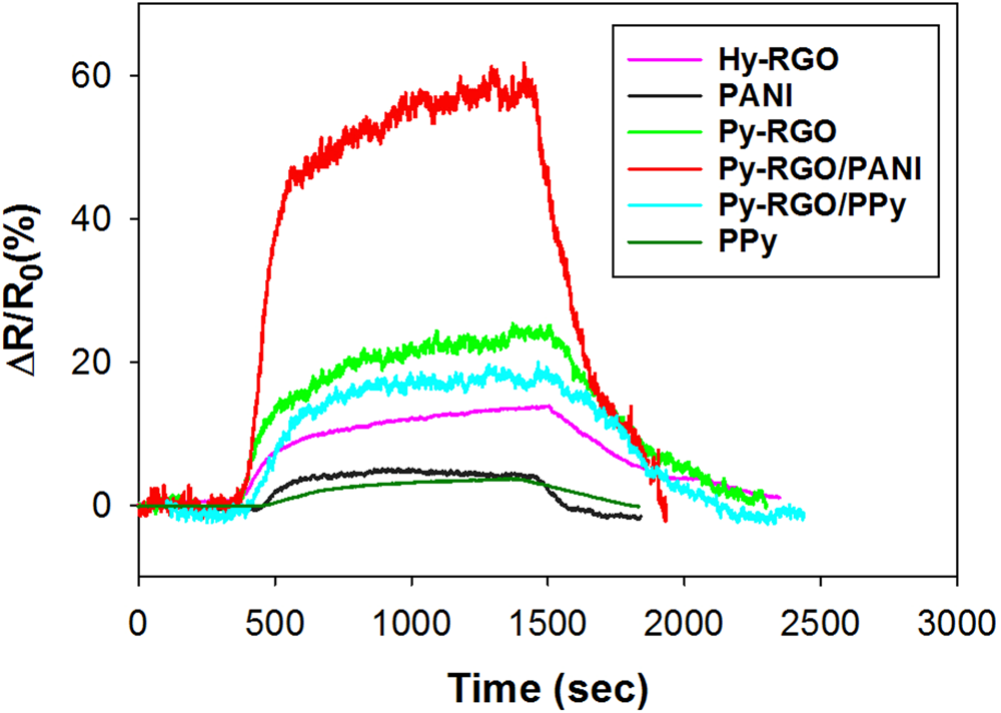
Comparison of the sensor responses for the LS films of Hy-RGO, Py-RGO, Py-RGO/PANI, and Py-RGO/PPy, and the casted films of PANI and PPy at an NH_3_ concentration of 50 ppm in a nitrogen environment.

The current-voltage (I–V) characteristics for the LS films deposited on the IDEs were found to be in the range of −5 to 5 V using the four-point method. As shown in Fig. 5, the sheet resistance for all the films remained almost a constant in the tested range, and this linear behavior is indicative of the ideal ohmic contact between the films and the electrodes. To check the reliability of the sensor, the response of the Hy-RGO film to 50 ppm NH_3_ gas was repeatedly measured in a nitrogen environment. The sensor’s response (ΔR/R_0_) upon exposure to NH_3_ gas was calculated by the following equation:

1 $${\rm{\Delta }}R/{R}_{0}=(\frac{{R}_{gas}-{R}_{0}}{{R}_{0}})\times 100\,( \% )$$

where *R*_0_ and *R*_*gas*_ are the electrical resistance values of the LS film-coated IDEs before and after exposure to NH_3_ gas in a nitrogen and/or an exhaled air environment, respectively. As shown in Fig. 6, the film exhibited a 14.8% response on average with 0.65% error upon three cycles of exposure to 50 ppm NH_3_ gas and evacuation. Six different films (i.e., four LS films and two casted films) were subjected to sensory response measurements at 50 ppm NH_3_ in a nitrogen environment, and the results are shown in Fig. 7. The order of response magnitude was Py-RGO/PANI ≫ Py-RGO > Py-RGO/PPy > Hy-RGO > PANI = PPy.

Importantly, the Py-RGO/PANI LS film exhibited the greatest sensory response, at about 59.1%, which was more than the responses of the other films. In general, RGO is well known for its p-type behavior with a high density of C=C sp^2^ carbon atoms acting as a hole-transporting matrix. When NH_3_ molecules adsorb to the RGO surface, they donate their lone pair electrons to the matrix due to the overlap between the highest occupied molecular orbitals (HOMO) of NH_3_ and the RGO orbitals^35^. The charge transfer results in the holes becoming filled and a concomitant increase in the resistance^24,36^. Although NH_3_ molecules interact with the Py-RGO surface by the same mechanism as for Hy-RGO, the Py-RGO surface has more sp^2^ carbon atoms due to Py oligomers, as discussed above. This is why the Py-RGO exhibited a response of 24.3%, which is greater than the 15.2% response for the Hy-RGO. The PANI prepared by *in-situ* polymerization is known to be a conductive form of emeraldine salt (ES)^28^. This ES has abundance of protons (H^+^) because it was doped with hydrochloric acid (1 M) in the preparation process, and the protons support electron transport throughout the polymer. When the NH_3_ molecules adsorb to the ES surface, they transformed the ES into an insulating emeraldine base by extracting protons to form ammonium ion^25^. Therefore, the exceptionally high response for the Py-RGO/PANI film can be attributed to the large increase in resistance due to the transformation into an insulating form. In addition, it appears that a film foming property of PANI was much better than that of PPy presumably because the RGO provided an adsorbent surface for PANI more efficiently than for PPy. Therefore, the better film foming property of PANI might be another contributor to such a high response of Py-RGO/PANI. On the other hand, the response for Py-RGO/PPy was 18.9%, which ranked 3^rd^ and which was much lower than that for Py-RGO/PANI. As PPy is also a p-type conductive polymer, the charge carriers in PPy are known to be polaron and bipolaron^37^. When electron-donating NH_3_ molecules adsorb to PPy, the concentration of the charge carriers decreases and thus the resistance increases due to electron transfer from NH_3_ to the π backbone of PPy^26,37^.

Lin *et al*. have proposed the following gas-sensing model of polypyrrole-poly(ethylene oxide) composite films:

2 $${\rm{\Delta }}R=({{\rm{r}}}_{1}-{{\rm{r}}}_{0})\frac{m}{n}\frac{{K}_{m}{C}_{0}}{1+{K}_{m}{C}_{0}}$$

where *ΔR*, *r*_0_, *r*_1_, *K*_*m*_, and *C*_0_ denote the difference between steady-state resistance before and after gas adsorption, the resistance of the vacant site, the resistance of the occupied site, the adsorption equilibrium constant, and the gas concentration^38,39^. They considered the overall resistance of the sensing film as *n* resistances of *R* in parallel and each *R* consists of *m* resistances of *r* in series. In other words, *R*, *r*, *n*, and *m* mean the resistance of a layer, the resistance of the site, the number of conduction paths, and the number of active sites in a monolayer. Therefore, a thinner sensing film with smaller *n* tends to produce a higher signal upon exposure to gas. This model can explain some of the response results in this study. The Py-RGO/PPy film had large PPy aggregates on its surface, resulting in a high *n* value, and it hence exhibited a relatively low response. On the other hand, the Py-RGO/PANI film had a relatively smooth surface with smaller aggregates (thus, a lower *n* value) but more active sites (thus, a higher *m* value) by the ES structure. In addition, this model can also explain why our LS films of Hy-RGO produced a much larger response (14.8% to 50 ppm NH_3_ gas) than the responses of RGO films reported in the literature, as they were thin, self-assembled films with well-controlled structures. For example, hydrazinium graphene spin-coated on IDEs produced an irreversible response of 0.5% to 5 ppm NH_3_^22^, and RGO film (reduced by NaBH_4_) dip-coated on IDEs resulted in a response of about 5% to 200 ppm NH_3_^23^. As another example, a less reversible response of about 30% was obtained when a three-dimensional (3D) graphene foam network prepared by chemical vapor deposition (CVD) was exposed to 1,000 ppm NH_3_^24^.

**Figure 8:**
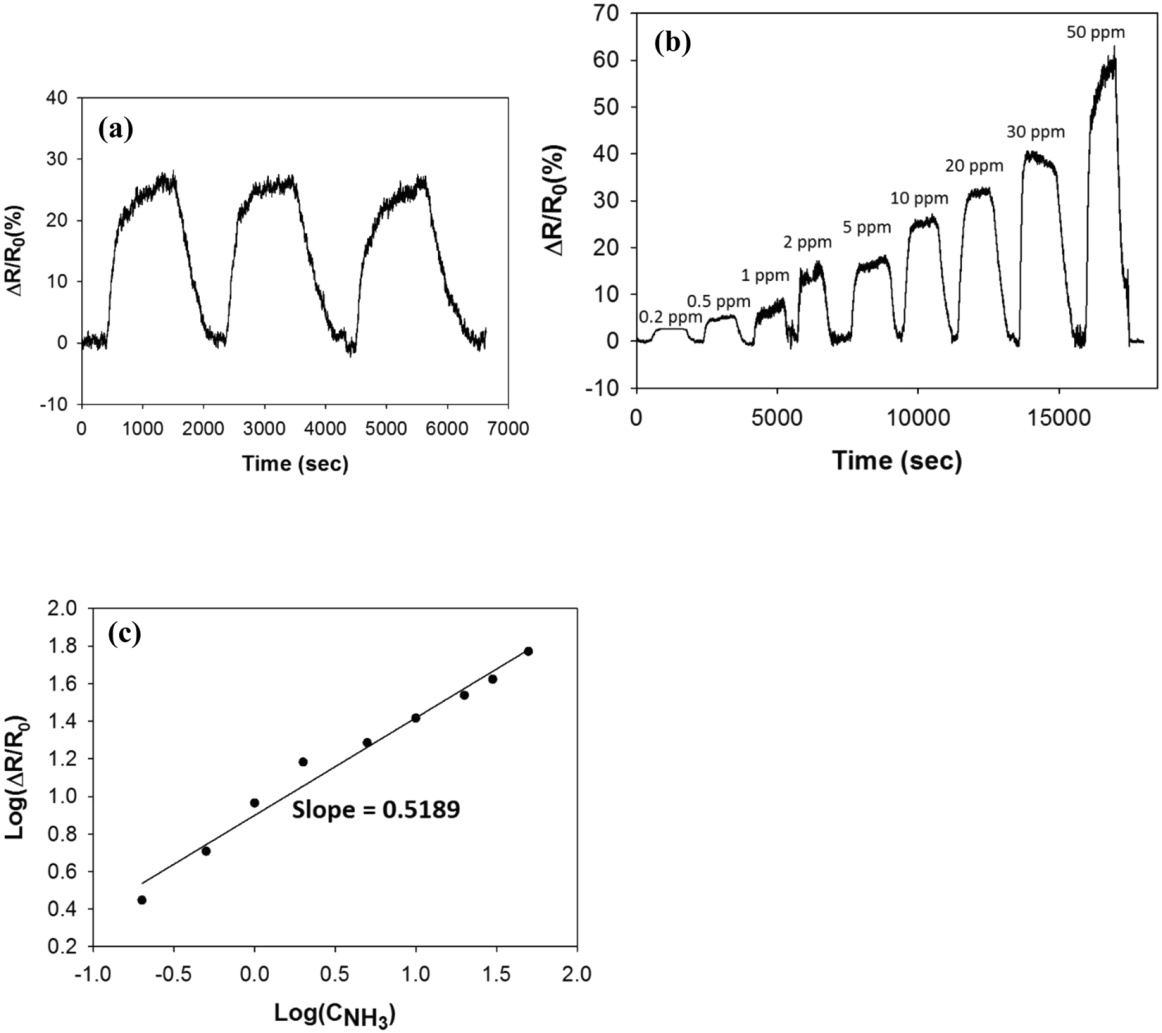
The sensor response for the LS films of Py-RGO/PANI in an N_2_ environment; (**a**) the reproducibility of the sensor response to 10 ppm NH_3_, (**b**) the concentration dependency of the sensor response from 0.2 to 50 ppm NH_3_ (**c**) sensitivity of the sensor response (r^2^ = 0.9784).

Figure 8 shows the sensor response for the Py-RGO/PANI film upon exposure to NH_3_. As shown in Fig. 8(a), during three cycles of exposure to 10 ppm NH_3_ gas and evacuation in an N_2_ environment, the film clearly exhibited a reproducible and strong response of about 26.4% on average, with 0.37% error, without further heat treatment. Figure 8(b) shows the concentration dependency of the sensor response of the Py-RGO/PANI film for a concentration range of 0.2 to 50 ppm. For many sensors with heterogeneous surfaces, concentration dependencies of power-law have been observed because the gas adsorption is not uniform across the surface^40,41^. Based on the Freundlich adsorption isotherm, the sensor response is related to the gas concentration by the following equation^40,41^:

3 $${\rm{\Delta }}R/{R}_{0}\propto (\frac{\alpha {C}^{\beta }}{1+\alpha {C}^{\beta }})$$

Where C, α and β denote the gas concentration, a proportionality factor, and the exponent. This equation is simplified to a power-law at a low concentration range^39^. The power-law relationship was applied to our data because the Py-RGO/PANI film was also heterogeneous, as discussed above. When the film was subsequently exposed to NH_3_ gas at concentrations from 0.2 to 50 ppm, the relationship between the logarithmic sensor response and the logarithmic concentration exhibit good linearity, with a slope = 0.52 and r^2^ = 0.9784 as shown in Fig. 8(c). Since no discernible response was obtained below a concentration of 0.2 ppm NH_3_, the lower limit of detection was taken to be 0.2 ppm. In addition, at the minimal detection limit (0.2 ppm), the response was about 2.8%, and thus the sensitivity was 14%/ppm. These results are better than those obtained by similar ammonia sensor studies in the literature, which is primarily attributed to synergistic effect of PANI and Py-RGO as discussed above. In addiiton, well-controlled structure of thin films fabricated by the LS technique is thought to enhance the response additionally by providing less conduction paths than those of thin films made by spin-coating, dip-coating and casting, etc. For example, Wu *et al*. fabricated drop-coated films of graphene and polyaniline nanocomposite, and they reported a detection limit of 1 ppm and a response of about 0.7% at 1 ppm. As shown in Table 1, compared to similar ammonia sensor studies, our results exhibited the lowest detection limit and the highest sensitivity at the detection limit, thus indicating that our thin films have better characteristics than the films described to date in the literature.

**Table 1:** Comparison of our ammonia-sensing results with those from other studies.

Materials	Sensor Response (%)	Detection Limit (ppm)	Sensitivity at Detection Limit (%/ppm)	Operating Temperature (°C)	Reference
RGO film	5.5 at 200 ppm 23 at 2800 ppm	200	0.0275	25	^24^
Graphene foam	3 at 20 ppm 20 at 1000 ppm	20	0.15	25	^23^
GO film	6 at 2 ppm 23 at 100 ppm	2	3	25	^49^
Nanostructured PANI film	5 at 5 ppm, 160 at 100 ppm	5	1	25	^50^
Graphene/PANI Film	0.7 at 1 ppm, 11.3 at 100 ppm	1	0.7	25	^51^
PPy film	1 at 4 ppm 31 at 100 ppm	1	1	25	^26^
SnO_2_ nanoparticles	30 at 50 ppm 305 at 1000 ppm	50	0.6	115	^52^
Py-RGO/PANI film	2.8 at 0.2 ppm 59.1 at 50 ppm	0.2	14	25	This work

**Figure 9:**
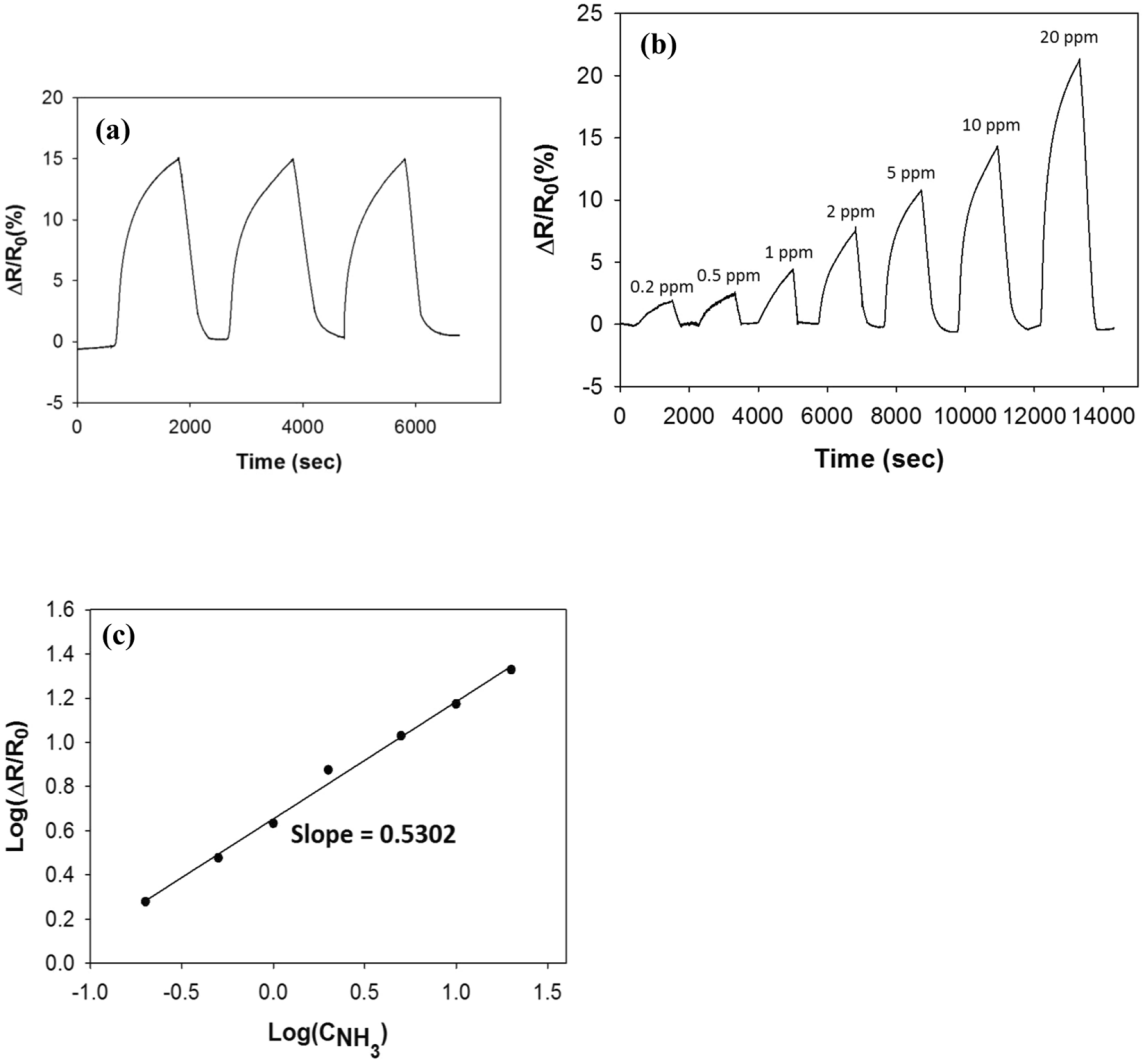
The sensor response for the LS films of Py-RGO/PANI in an exhaled air environment; (**a**) reproducibility of the sensor response to 10 ppm NH_3_ (**b**) the concentration dependency of the sensor response to the range of 0.2 to 20 ppm NH_3_ (**c**) sensitivity of the sensor response (r^2^ = 0.9944).

Figure 9 shows the sensor response results for simulated exhaled air. Again, a good response reproducibility was obtained, with an average response of about 14.9% and 0.14% error at 10 ppm NH_3_. Also, sensitivity behaviors similar to those in nitrogen were observed: a good linear sensitivity, with a slope of 0.53 and r^2^ = 0.9944. The response level for exhaled air decreased to about a half in nitrogen (for example, the responses to 10 ppm NH_3_ were 14.9% for exhaled air and 26.4% for nitrogen), presumably due to competitive adsorption of other components such as electron-accepting CO_2_. However, the overall response remained reproducible and, proportional to the concentration, with good linearity and high sensitivity, and the sensitivity slopes, in particular, remained nearly the same. These results indicate that the Py-RGO/PANI film has a reasonable selectivity toward NH_3_, even at the very low concentrations in exhaled air. As discussed above, the NH_3_ sensing mechanism of the Py-RGO/PANI, i.e., the hole-filling and the deprotonation, operates only for the adsorption of NH_3_ molecules and not electron-accepting CO_2_ because the adsorption of electron-accepting CO_2_ molecules increases the hole density and thereby results in a reduced resistance^42^. One distinct difference is that the noise level in exhaled air was much lower than that for a N_2_ environment, which was presumably due to competitive adsorption with other components such as CO_2_. These results indicate that our sensor films have the potential for use in the diagnosis of renal disease (and possibly also peptic ulcers), which requires a typical detection range of 1.5 to 15 ppm NH_3_ (and 0.05 to 2 ppm NH_3_ for peptic ulcers) in exhaled air.

In the past several decades, several different types of NH_3_ sensors have been developed, but their limitations have hampered their practical use for diagnostic purpose. For example, while sensors based on metal oxide and catalytic metal are known to have a minimal detection limit as low as 1 ppm, they suffer from low selectivity and the need for high operating temperatures (>400 °C)^8^. In addition, optical gas sensors such as coulometric and adsorption spectroscopy are ultrasensitive for the detection of NH_3_ at concentrations down to 1 ppb, but these sensors require large and expensive instruments^43,44^. By contrast, our sensor based on Py-RGO/PANI thin films appears to be promising as a prototype sensor for diagnostic purposes in light of its facile fabrication, high sensitivity, portability, ease of use at room temperature, and low price.

## Conclusions

We prepared four different types of reduced graphene particles and their conductive polymer-hybrid particles: Hy-RGO, Py-RGO, Py-RGO/PANI, and Py-RGO/PPy, and we fabricated four corresponding thin films with well-controlled thicknesses and structures on interdigitated electrodes using LS techniques. Ammonia-sensing experiments with these thin films revealed that the Py-RGO/PANI film exhibited the highest level of sensor response, presumably because it had the most advantageous structural characteristics among the samples that we tested. The film also yielded a high level reproducibility, high linearity at low concentrations, and a minimal detection limit as low as 0.2 ppm both in N_2_ and exhaled air environments. In addition to these desirable gas-sensing properties, in light of further advantages such as facile fabrication, ease of use at room temperature, portability, and low price make this type of sensor a promising tool for the diagnosis of renal disease.

## Experimental Section

### Materials

Graphite flakes (+100 mesh), pyrrole (reagent grade, 98%), aniline (ACS reagent, 99.5%), N,N-Dimethylformamide (DMF, anhydrous, 99.8%), ammonium persulfate (APS, ACS reagent, >98%), hydrazine monohydrate (reagent grade, 98%), ethanol (anhydrous, 99.9%) and hydrogen peroxide (ACS reagent, 30%) were purchased from Sigma-Aldrich and used without further purification. Deionized (DI) water (18.2 MΩ•cm) was used in all of the experiments. Oxidized silicon (Si/SiO_2_, Silicon Technology Inc., Salt Lake City, USA) wafers were used as substrates. Pure air, nitrogen, and ammonia gases were purchased from Dong-A Specialty Gases Corp (Seoul, South Korea).

### Reduction of graphene oxide by hydrazine (Hy-RGO) and pyrrole (Py-RGO)

Graphene oxide (GO) was synthesized from graphite according to a modified version of the Hummers’ method^45^. The GO powders were dispersed in DI water at a concentration of 3 mg/ml. Prior to reduction, 4 ml of the as-prepared GO suspension was mixed with 36 ml of DMF, which results in a DMF/DI water solvent system (9:1 vol/vol) for obtaining a stable suspension^46^. The hydrazine solution was then added to the suspension under vigorous stirring, and the reduction reaction was carried out in a jacketed beaker at 80 °C for 12 h. At the end of the reaction, the suspension color turned entirely black. 3 ml of hydrogen peroxide was added to neutralize the excess hydrazine, thereby resulting in the hydrazine-reduced graphene oxide (Hy-RGO) suspension.

The GO was also reduced by pyrrole^47^. Similarly, 9 ml of the GO suspension was dispersed in 21 ml of DI water, followed by sonication for 10 min. Pyrrole (6 ml) was dissolved in 30 ml of ethanol and mixed with the GO suspension. The mixture was sonicated for additional 1 h. The reduction was carried out in a round-bottom flask connected to a condenser with circulating water at 95 °C for 12 h. After the reaction, a black suspension was obtained that was filtered, and washed thoroughly using DMF and ethanol. The Py-RGO powders were then collected and dried at ambient condition overnight.

### Synthesis of PANI and PPy

PANI and PPy were synthesized by standard procedures using APS as an initiator^26,28,29^. For the preparation of PANI, 3 ml of aniline was dissolved in 100 ml of 1 M HCl aqueous solution with sonication for 30 min. The HCl solution (20 ml) containing 7 g APS was then added dropwise to the aniline solution under vigorous stirring, and the polymerization reaction was carried out at 4 °C for 10 h. The resulting polymer solution was filtered, washed with copious amounts of DI water, ethanol, and hexane, and then dried at 60 °C for 12 h. The resulting green PANI powders were collected. For the preparation of PPy, 1 ml of pyrrole was dissolved in 75 ml of DI water by sonication for 30 min. An aqueous solution of 4 g APS (25 ml) was added dropwise to the pyrrole solution under vigorous stirring, and the polymerization reaction was carried out at 4 °C for 10 h. The polymer solution was then filtered, washed with copious amounts of DI water and ethanol, and dried as before, thereby resulting in black PPy powders.

### Synthesis of Py-RGO/PANI and Py-RGO/PPy

Hybrid Py-RGO/PANI and/or Py-RGO/PPy powders were prepared by *in-situ* polymerization of PANI and/or PPy in the presence of Py-RGO, using the procedures described previously. For the Py-RGO/PANI, 10 mg of Py-RGO was dispersed in 10 ml of 1 M HCl aqueous solution by sonication for 30 min. Aniline (25 µl) and APS (65 mg) were added to the dispersion. Similarly, for the Py-RGO/PPy, 10 mg of Py-RGO was dispersed in 10 ml of DI water, and 16.5 μl of pyrrole and 66.7 mg of APS were added to the dispersion. This resulted in a greenish-black powder of Py-RGO/PANI and a black powder of Py-RGO/PPy.

### Preparation of Sensor Films by the Langmuir-Schaefer (LS) Technique and Casting

Interdigitated microelectrodes (IDEs) were custom-fabricated using a combined process of sputtering and lithography by a company (J-solution, Seoul, South Korea). First, a thin film of Ti (20 nm thickness) was coated on the top of an oxidized silicon wafer, and a thin Cu layer (50 nm thickness) was subsequently formed on the top of the Ti film by sputtering. The interdigitated structure was then fabricated by a typical process of positive photoresist lithography and wet etching. In order to prepare the Langmuir-Schaefer (LS) films, ethanol suspensions of specific RGO or hybrid powders were prepared at concentrations of 0.3 mg/ml, and they were spread on the water subphase. The spread films were compressed by control barriers at a speed of 10 mm/min, and the LS films of Hy-RGO, Py-RGO, Py-RGO/PANI, and Py-RGO/PPy were transferred onto the IDEs horizontally at a surface pressure of 30 mN/m. For comparison, PANI and PPy films were also prepared by film-casting the ethanol dispersions of the corresponding polymers at a concentration of 1 mg/ml on IDEs. All of the films were dried at 0 °C for 1 h and securely stored under vacuum for the gas-sensing measurements.

### Characterization

Raman spectroscopy (Xper Ram 200, NanoBase) and Fourier-transform infrared spectroscopy (FT-IR, Bruker Optik, 3000) were used to examine the surface structure and the chemical composition of the LS films. The morphology and surface roughness of the LS films were measured using field emission scanning electron microscopy (FE-SEM, JSM-7100F, JEOL), and atomic force microscopy (AFM, N8 NEOS, Bruker), respectively. The structures of the composites were examined by high-resolution transmission electron microscopy (HR-TEM, JEM-300F; JEOL Ltd.), and their chemical compositions were determined by X-ray photoelectron spectroscopy (XPS, K-alpha; Thermo Scientific Inc.). Thermogravimetric Analysis (TGA, Pyris 1 TGA, PerkinElmer) was employed to study thermal stability of the composites.

### Ammonia-sensing measurement

All of the ammonia-sensing measurements were conducted at room temperature in a gas chamber (P7000; Made Lab) equipped with a computer-interfaced multi-channel source meter (2400, Keithley) and mass flow controllers (MFC, MPR-3000S, MFC Korea). First, the LS film-deposited IDEs were positioned in a nitrogen environment at 1 atm to obtain a stable baseline, and they were then exposed to NH_3_ gas at a well-controlled concentration in a nitrogen environment. In order to mimic exhaled breath, we prepared simulated breath composed of 76.3% nitrogen, 15.2% oxygen, 4% carbon dioxide, 3.1% water vapor, 0.83% argon, and small amounts of neon and helium by mixing air with carbon dioxide using an MFC. This composition is close to that of typical exhaled breath^48^. For ammonia-sensing measurements, the prepared exhaled air was first injected into the chamber, the baseline was checked, and an NH_3_ stream at the desired concentration was introduced. The change in the resistance upon the exposure to NH_3_ with a four-point connection supplying a constant current of 500 μA was then monitored using the multi-channel source meter. After the exposure to NH_3_ gas for a constant period of time (about 1,100 s), the chamber was flushed with a flow of fresh nitrogen or exhaled air to purge the existing gas mixture, and it was refilled with new gas at another concentration.

## Electronic supplementary material

Highly sensitive ammonia sensor for diagnostic purpose using reduced graphene oxide and conductive polymer

## Data Availability

All data generated or analysed in this study are included in this article.
