# Supplementary material for: Highly sensitive ammonia sensor for diagnostic purpose using reduced graphene oxide and conductive polymer
Source: Sci Rep. 2018 Dec 21;8:18030. doi: 10.1038/s41598-018-36468-z (PMC6303394; doi:10.1038/s41598-018-36468-z)
Supplement: Supplementary file 1 — Highly sensitive ammonia sensor for diagnostic purpose using reduced graphene oxide and conductive polymer [file 41598_2018_36468_MOESM1_ESM.docx]

**Title:** Highly sensitive ammonia sensor for diagnostic purpose using reduced graphene oxide and conductive polymer

Tan Nhiem Ly and Sangkwon Park*

Tan Nhiem Ly; Prof. Sangkwon Park

Department of Chemical and Biochemical Engineering, Dongguk University

Pildong-ro 1-gil 30, Jung-gu, Seoul, 04620, South Korea.

**(b)**

**(a)**

**(c)**

Figure S1. XPS spectra of Py-RGO, Py-RGO/PANI, and Py-RGO/PPy.

The C1s, N1s, and O1s XPS spectra of the hybrid films are shown in Fig. S1. For all the hybrid films, the C1s spectra apparently show large peaks at around 284-285 eV, the N1s spectra do at about 398-399 eV, and the O1s spectra at about 531-532 eV. According to studies in the literature, the C 1s peaks for Py-RGO and/or RGO/PANI hybrid films contain deconvoluted peaks at about 284, 286, and 288 eV, which are attributed to C-C/C=C bonds of the sp^2^ aromatic carbon structures, C-O bonds of the carbonyl and epoxy groups, and C=O bonds of the carboxyl groups, respectively^1,2^ .Similarly, the N 1s spectra have deconvoluted peaks at about 398, 399 and 401 eV, which correspond to quinoid amine, benzenoid amine, and nitrogen cationic radical, respectively^1,3^ .The O 1s spectra contain deconvoluted peaks at about 530 and 532 eV, which are for C=O and OH-C=O groups, and C-OH groups, respectively^4^ .

From the XPS spectra, the N atomic percentages were estimated to be 10.83%, 14.86%, and 12.31% for Py-RGO, Py-RGO/PANI, and Py-RGO/PPy, respectively, and the O atomic percentages were 12.45%, 14.23%, and 10.6% for Py-RGO, Py-RGO/PANI, and Py-RGO/PPy, respectively. The Py-RGO/PANI and Py-RGO/PPy films showed higher N atomic percentages than that for Py-RGO obviously because of PANI and PPy molecules existing at the Py-RGO surface. In addition, the Py-RGO/PANI film showed the highest content of oxygen and a larger O1s peak shifted at 533 eV presumably because water molecules were adsorbed on the protonated ES form of PANI^5^ .

Figure S2. TGA curves of Py-RGO, Py-RGO/PANI, and Py-RGO/PPy.

Thermogravimetric analysis (TGA) curves are shown in Fig. S2. Overall, similar thermal degradation behaviors were observed for all the hybrid films; Up to 100 ⁰C, there was an initial weight loss of about 6% due to evaporation of adsorbed water molecules^4^ , then more weight loss up to around 650 ⁰C (e.g. 30.8% at 660 ⁰C for Py-RGO/PANI), and a significant weight loss due to decomposition of PPy, PANI and RGO after around 650 ⁰C. For Py-RGO/PANI, a slightly less durable behavior was observed in the range of 280 to 660 ⁰C presumably due to the deprotonation of PANI^6^ .

**References**

1. Zhu, J. *et al.* The effect of various electrolyte cations on electrochemical performance of polypyrrole/RGO based supercapacitors. *Phys. Chem. Chem. Phys.* **17,** 28666–28673 (2015).
2. Wu, Z. *et al.* Enhanced sensitivity of ammonia sensor using graphene/polyaniline nanocomposite. *Sens. Actuators B Chem.* **178,** 485–493 (2013).
3. Zhang, K., Zhang, L. L., Zhao, X. S. & Wu, J. Graphene/polyaniline nanofiber composites as supercapacitor electrodes. *Chem. Mater.* **22,** 1392–1401 (2010).
4. Akhavan, O. The effect of heat treatment on formation of graphene thin films from graphene oxide nanosheets. *Carbon* **4 8**, 509 –519 (2010).
5. Chen, G., Lu, Q. F., Zhao, H. B. SnO_2_-Decorated graphene/polyaniline nanocomposite for a high-performance supercapacitor electrode. *J. Mater. Sci. Technol.* **31***,* 1101–1107 (2015)
6. Yan, L., Rui, J. D., Zan, W., & Liu, H. T. Carboxyl-functionalized graphene oxide–polyaniline composite as a promising supercapacitor material. *J. Mater. Chem*. **22**, 13619–13624 (2012).
